# Diet at birth is critical for healthy growth, independent of effects on the gut microbiota

**DOI:** 10.1186/s40168-024-01852-7

**Published:** 2024-07-27

**Authors:** Lieke J. W. van den Elsen, Akila Rekima, Miriam A. Lynn, Charlotte Isnard, Savannah Machado, Nivedithaa Divakara, Diana Patalwala, Alana Middleton, Natalie Stevens, Florence Servant, Remy Burcelin, David J. Lynn, Valerie Verhasselt

**Affiliations:** 1 Larsson-Rosenquist Centre for Immunology and Breastfeeding, School of Medicine, The University of Western Australia, Perth, WA Australia; 2 Telethon Kids Institute, Perth, WA Australia; 3 South Australian Health and Medical Research Institute, Adelaide, SA Australia; 4 University of Nice, Nice, France; 5 National Imaging Facility, Centre for Microscopy Characterisation and Analysis, University of Western Australia, Perth, WA Australia; 6 Vaiomer, Toulouse-Labège, France; 7 Inserm 1297, I2MC, Toulouse, France; 8 Flinders Health and Medical Research Institute, Flinders University, Adelaide, SA Australia

**Keywords:** Growth hormone resistance, Neonatal microbiota, Growth failure, Breast milk, Colostrum

## Abstract

**Background:**

Colostrum is the first milk for a newborn. Its high content in microbiota shaping compounds and its intake at the time of gut microbiota seeding suggests colostrum may be critical in the establishment of a healthy microbiota. There is also accumulating evidence on the importance of the gut microbiota for healthy growth. Here, we aimed to investigate the contribution of colostrum, and colostrum-induced microbiota to growth promotion. Addressing this question is highly significant because (1) globally, less than half of the newborns are fully colostrum fed (2) the evidence for the importance of the microbiota for the prevention of undernutrition has only been demonstrated in juvenile or adult pre-clinical models while stunting already starts before weaning.

**Results:**

To address the importance of diet at birth in growth failure, we developed a unique mouse model in which neonates are breastfed by mothers at an advanced stage of lactation who no longer provide colostrum. Feeding newborn mice with mature milk instead of colostrum resulted in significant growth retardation associated with the biological features of chronic undernutrition, such as low leptin levels, dyslipidemia, systemic inflammation, and growth hormone resistance. We next investigated the role of colostrum in microbiota shaping. At the end of the lactation period, we found a major difference in gut microbiota alpha diversity, beta diversity, and taxa distribution in control and colostrum-deprived mice. To determine the causal relationship between changes in microbiota and growth trajectories, we repeated our experiment in germ-free mice. The beneficial effect of colostrum on growth remained in the absence of microbiota.

**Conclusion:**

Our data suggest that colostrum may play an important role in the prevention of growth failure. They highlight that the interplay between neonatal gut microbiome assembly and diet may not be as crucial for growth control in the developing newborn as described in young adults. This opens a paradigm shift that will foster research for colostrum’s bioactives that may exert a similar effect to microbiota-derived ligands in promoting growth and lead to new avenues of translational research for newborn-tailored prevention of stunting.

Video Abstract

**Supplementary Information:**

The online version contains supplementary material available at 10.1186/s40168-024-01852-7.

## Background

In 2020, globally 200 million children under 5 years of age were estimated to be undernourished [1]. About half of the deaths among children under 5 are linked to undernutrition, resulting in over 3 million deaths per year, mostly affecting low- and middle-income countries (LMIC) [1]. In addition to playing a major role in the burden of child mortality, chronic undernutrition leads to abnormal development such as growth failure (small for age or stunting), immune dysfunction and neurodevelopment deficits that are irreversible by the age of two [1–3]. Identifying early interventions to prevent undernutrition is thus a pressing goal, with important and lasting health, economic and social impacts for individuals and their families, communities and countries [1]. In the last decade, the gut microbiota has become a key target for strategies aiming at controlling energy metabolism and weight gain. Many studies have revealed that specific gut microbes can both promote obesity [4] and play a causal role in severe acute underweight individuals [2, 5]. In addition, recent evidence has been accumulating on the importance of gut microbiota for juvenile linear growth [2, 6–9]. While these data have highlighted the importance of the interplay between the diet and gut microbiota for the regulation of growth and weight gain post-weaning, there is a striking gap in knowledge on the importance of this crosstalk before weaning. Filling this gap is important because stunting develops during this early period of life [3]. In this study, we aimed to address this question by looking at the role of colostrum in the promotion of healthy growth through the seeding and shaping of the gut microbiota. Colostrum is the first breastmilk a newborn should receive within one hour after birth and is produced during the first 2–3 days of life [10]. We hypothesised that the crosstalk between colostrum and the gut microbiota is critical for healthy growth before weaning. This hypothesis is based on the timing of colostrum intake (at birth) when the gut becomes colonised, and on its content of high levels of microbiota-shaping compounds such as IgA, lactoferrin and human milk oligosaccharides compared to mature milk and even more so to pre-lacteal feeds such as formula [10–14]. In addition, colostrum itself has a microbiota composition that is distinct from mature milk and typically absent from pre-lacteal feeds [15–17]. We therefore expect the specific composition of colostrum to match the specific needs of the developmental age of the newborn, including the sequential establishment of the microbiota. Importantly, more than 50% of the newborns in LMIC are not optimally colostrum-fed due to delayed breastfeeding initiation, colostrum withdrawal and/or pre-lacteal feed administration [18, 19]. This suggests that colostrum may be a missing link required to establish a microbiota that empowers the newborn to resist being underweight. To approach this question, we have begun to develop a preclinical model of colostrum deprivation to evaluate the need for colostrum for healthy growth, and the contribution of change in microbiota in growth promotion.

## Results

### Colostrum deprivation at birth causes growth failure in mice

**Fig. 1:**
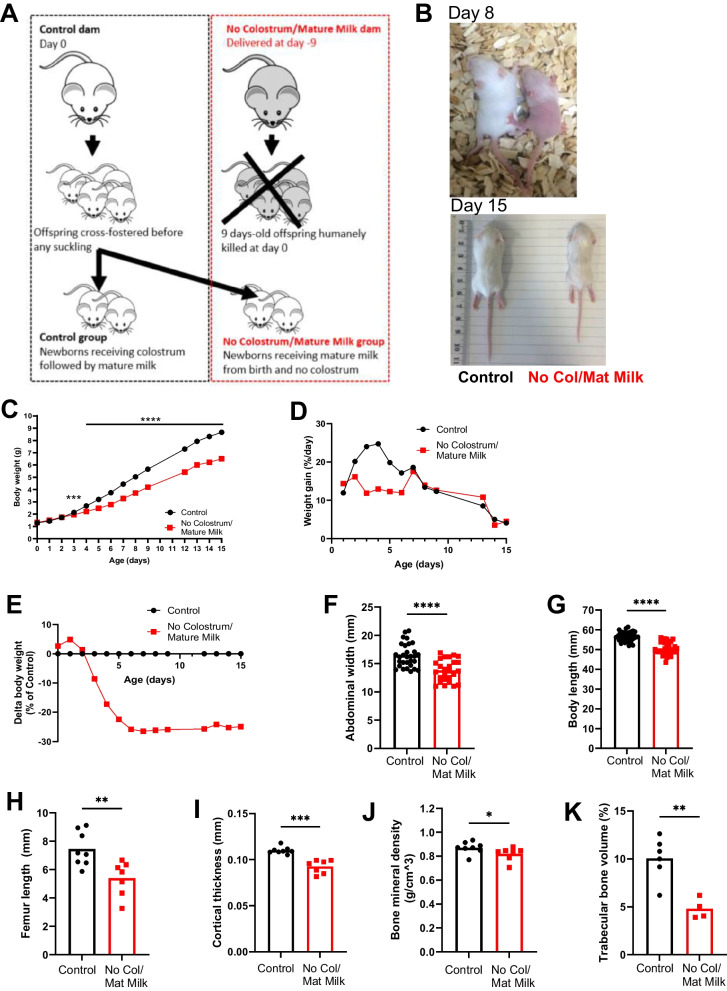
Colostrum deprivation at birth causes growth failure in mice. **A** Preclinical model of colostrum deprivation/mature milk feeding from birth. Pups were fostered at birth to a dam providing colostrum followed by mature milk (Control group (black), physiologically breastfed) or a dam that gave birth 9 days prior and not providing colostrum anymore but mature milk (No Colostrum/mature milk group, No Col/Mat milk (red)). **B** Photograph of representative mice of the Control and No Colostrum/Mature milk group at days 8 and 15. **C** Body weight growth curve, (**D**) growth rate expressed as percentage weight gain per day and (**E**) body weight as a percentage of the control group. **E** abdominal width and (F) Body length at day 15. **H** femur length determined using callipers on day 15. Micro-CT analysis of femur bones at day 15 was used to determine (**I**) cortical thickness, (**J**) cortical bone mineral density and (K) trabecular bone volume as a percentage of the total volume of interest. Data are presented as means with individual values or means ± SEM; 5 experiments with *n* = 6–13 per group (**A**,**B**,**C**,**D**); 4 experiments with 5–12 mice per group ( **E**,**F**) and 1 experiment with *n* = 7–8 per group (**H**,**I**, **J**) and with 4–6 mice per group (**K**). Statistical analysis of the difference between Control and No Colostrum/Mature milk groups was performed using Mann–Whitney test. *P < 0.05, **P < 0.01, ***P < 0.001, ****P < 0.0001

We first aimed to establish whether the diet at birth plays a causal role in underweight susceptibility in early post-natal life. Much like human milk, mice have different lactation stages with colostrum being produced first followed by mature milk [20–23] (Fig. S1). Therefore, cross-fostering pups immediately after birth to a dam that was already at an advanced stage of lactation and comparing their growth to control pups who were physiologically nursed (and also cross-fostered to control dams to account for possible stress-induced developmental changes), allowed us to evaluate the importance of colostrum for growth (Fig. 1A). Feeding newborns from birth with mature milk instead of colostrum severely affected the development of infant mice as shown on representative photography at day 8 and 15 (Fig. 1B). Control pups gained weight during the pre-weaning period (Fig. 1C), with the percentage weight gain per day peaking at 25% on days 3 and 4 of life (Fig. 1D). In contrast, pups that did not consume colostrum at birth were not able to accelerate weight gain during this time window and demonstrated significantly lower body weights compared to control pups from day 3 of life onwards (Fig. 1C, D). The difference in body weight between the groups reached a plateau from day 6 after which the body weight in the No Colostrum/mature milk group remained 25% lower than the Controls (Fig. 1E). Mice deprived of colostrum also had a significant decrease in abdominal width and body length at 2-weeks-old (Fig. 1F, G), a characteristic of chronic undernutrition [1]. Growth failure was associated with decreased bone development, as demonstrated by shorter femur bones (Fig. 1H), and reduced cortical bone thickness, cortical bone mineral density and trabecular bone volume (Fig. 1I-K, S1B).

**Fig. 2:**
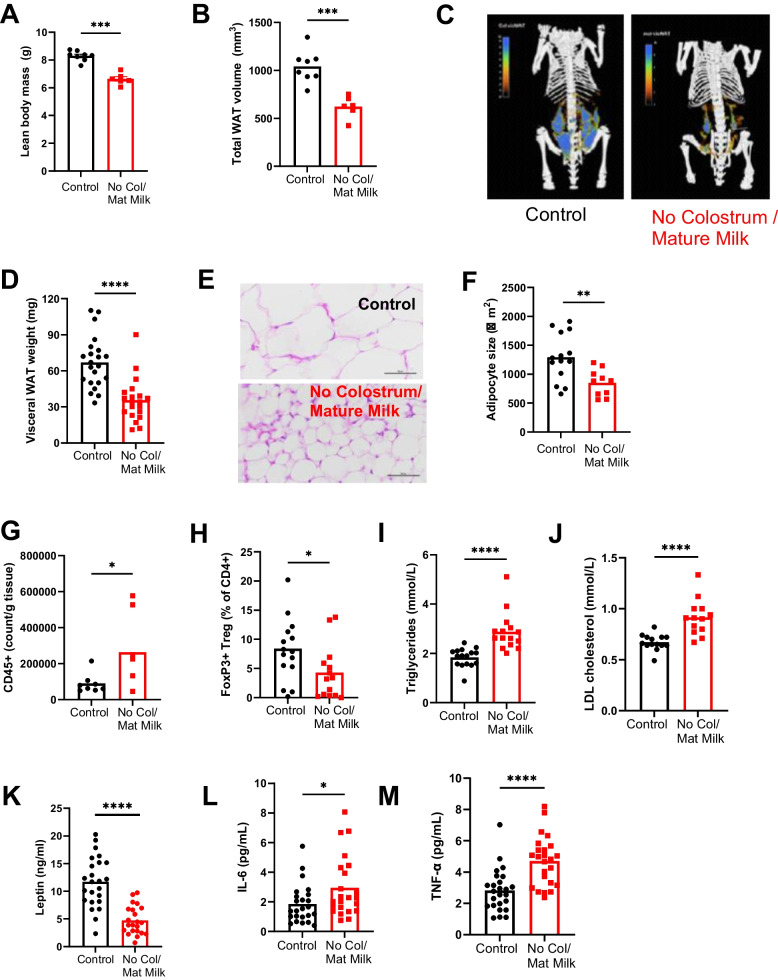
Mice reared without colostrum are undernourished at 2 weeks of age. Micro-CT analysis of live mice reared with or without colostrum at 2 weeks old demonstrating (**A**) lean body mass and (**B**) the total white adipose tissue (WAT) volume. **C** Representative micro-CT scan images depicting the visceral WAT. **D** Weight of visceral WAT. **E** Representative image of hematoxylin and eosin-stained gonadal WAT (400 × magnification, scale bar 50 µm). **F** Average gonadal WAT adipocyte area. **G** CD45 + cells per gram of adipose tissue (**H**) Percentage of FoxP3 + Treg cells among CD4 + T cells. Concentration of (**I**) triglycerides, (**J**) LDL) cholesterol, (**K**) leptin, (**L**) TNF-α and (M) IL-6 in plasma. Data are presented as means with individual values. One experiment with n = 6–8 per group (**A**, **B**), 3 experiments with n = 5–10 (D), *n* = 3–5 (**F**), *n* = 6–8 (H), *n* = 4–6 (I, J) and *n* = 7–9 (**K**, **L**, **M**) per group. 2 experiments with 6–7 per group (**G**). Statistical analysis of the difference between Control and No Colostrum/Mature milk groups was performed using Mann–Whitney test. *P < 0.05, **P < 0.01, ***P < 0.001, ****P < 0.0001

In addition to being short-for-age, mice lacking colostrum at birth showed reduced abdominal widths (Fig. 1F), suggesting thinness. Micro-CT analysis of 2-week-old mice demonstrated that the lean body mass (Fig. 2A) as well as the white adipose tissue (WAT) volume (Fig. 2B) were significantly reduced. The amounts of both visceral (Fig. 2C, D, S1C) and subcutaneous (Fig. S1D) WAT were severely reduced in the absence of colostrum feeding, even after correction for total body volume (Fig. S1C), as was the adipocyte size (Fig. 2E, F). In addition to impaired adipose tissue development, we observed adipose tissue immune dysregulation. Mice lacking colostrum intake at birth but fed mature milk demonstrated a higher number of CD45^+^ leukocytes in the adipose tissue (Fig. 2G). Importantly, when we looked at the percentage of FoxP3^+^ regulatory T cells (Treg) (Fig. 2H), which are critical in the control of energy metabolism, these were significantly reduced in the No Colostrum/mature milk group as compared to controls. The percentage of type 2 innate lymphoid cells, which also play a role in AT homeostasis, was similar in Control versus No Colostrum/mature milk mice (data not shown). Abnormal lipid metabolism in mice lacking colostrum at birth was further observed in blood lipids levels. Plasma triglycerides (Fig. 2I) and low-density lipoprotein (LDL) cholesterol (Fig. 2J) were increased at 2 weeks of age if mice were reared without colostrum, while total cholesterol (Fig. S1E) and high-density lipoprotein (HDL) cholesterol (Fig. S1F) were similar in both groups. Lastly, we observed that other biological hallmarks of chronic undernutrition [24], i.e. low plasma leptin (Fig. 2K) and low-grade inflammation marked by increased proinflammatory cytokines Tumour Necrosis Factor (TNF)-alpha and Interleukin (IL)-6 (Fig. 2L, M) in plasma, were also present in No Colostrum/Mature milk mice. Altogether, these data show that colostrum deprivation at birth in exclusively (mature milk) breastfed mice was sufficient to reproduce many of the findings of chronic undernutrition. At the end of the lactation period, mice were underweight and small for their age and presented with both developmental and metabolic abnormalities. Importantly, as observed in the human setting [1], abnormal development was irreversible as underweight and reduced height persisted into adulthood (Fig. S2A-C).

### Lack of colostrum leads to growth hormone resistance

**Fig. 3:**
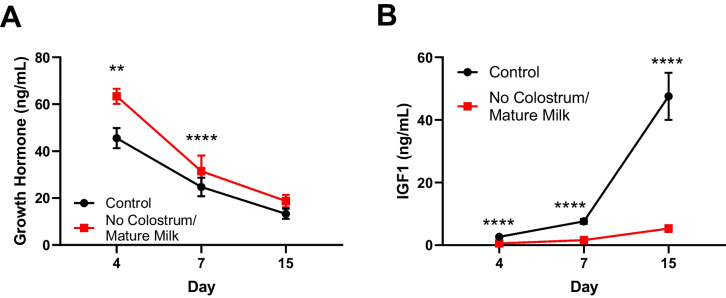
Lack of colostrum leads to growth hormone resistance. **A** Growth hormone and (**B**) insulin-like growth factor 1 (IGF-1) in plasma from mice reared with and without colostrum over time. Data are presented as means with individual values depicted or means ± SEM. 1 experiment for day 4 with *n* = 8–9 per group; 2 experiments for day 7 with *n* = 5–8 per group; 3 experiments for day 14 with *n* = 7–8 per group. Statistical analysis was performed using Mann–Whitney test. *P < 0.05, **P < 0.01, ***P < 0.001, ****P < 0.0001

Growth failure in mice deprived of colostrum at birth suggested alteration of the somatotropic axis, a key endocrine mechanism regulating postnatal growth [24, 25]. Growth hormone (GH) is secreted by the anterior pituitary gland and stimulates the production of insulin-like growth factor-1 (IGF-1), primarily by the liver. IGF-1 promotes organ and systemic growth. We, therefore, measured circulating GH and IGF-1 in the plasma of mice reared with and without colostrum. Feeding mature milk at birth instead of colostrum severely altered the activity of the somatotropic axis as shown by high to normal plasma concentrations of growth hormone (Fig. 3A) and severely reduced plasma IGF-1 (Fig. 3B) concentrations, characteristic of growth hormone resistance [24, 25].

The main factors responsible for GH resistance are undernutrition and chronic infection [24–26]. The normal to high weight gain by the No Colostrum/Mature milk group during the first 2 days indicated that pups were well fostered by their dams. This was further confirmed by similar milk intake in both groups (Fig. S3A). We then evaluated whether colostrum-deprived/mature milk fed mice had a deficit in macronutrient intake. We found a lower fat content and higher protein content in colostrum collected on day 1 compared to milk collected at a later stage. However, at the time of onset of growth failure (day 3–4), we found no significant difference in macronutrient content ( milk from days 3–4 for the control and days 12–13 for the no colostrum group) (Fig. S1A). Small intestine length (Fig. S3B) and villus lengths (Fig. S3C) were similar in 4- and 14-days-old mice reared with and without colostrum at birth, demonstrating no major alterations in gut absorptive capacity by colostrum deprivation. Loss of energy due to lipid malabsorption was excluded as the percentage of lipids in the faeces was similar between both groups of mice (Fig. S3D). Finally, as these mice were specific-pathogen-free (SPF) and raised in individually ventilated cages, we excluded infection as a potential cause of GH resistance.

### Growth retardation in colostrum-deprived mice is microbiota-independent

**Fig. 4:**
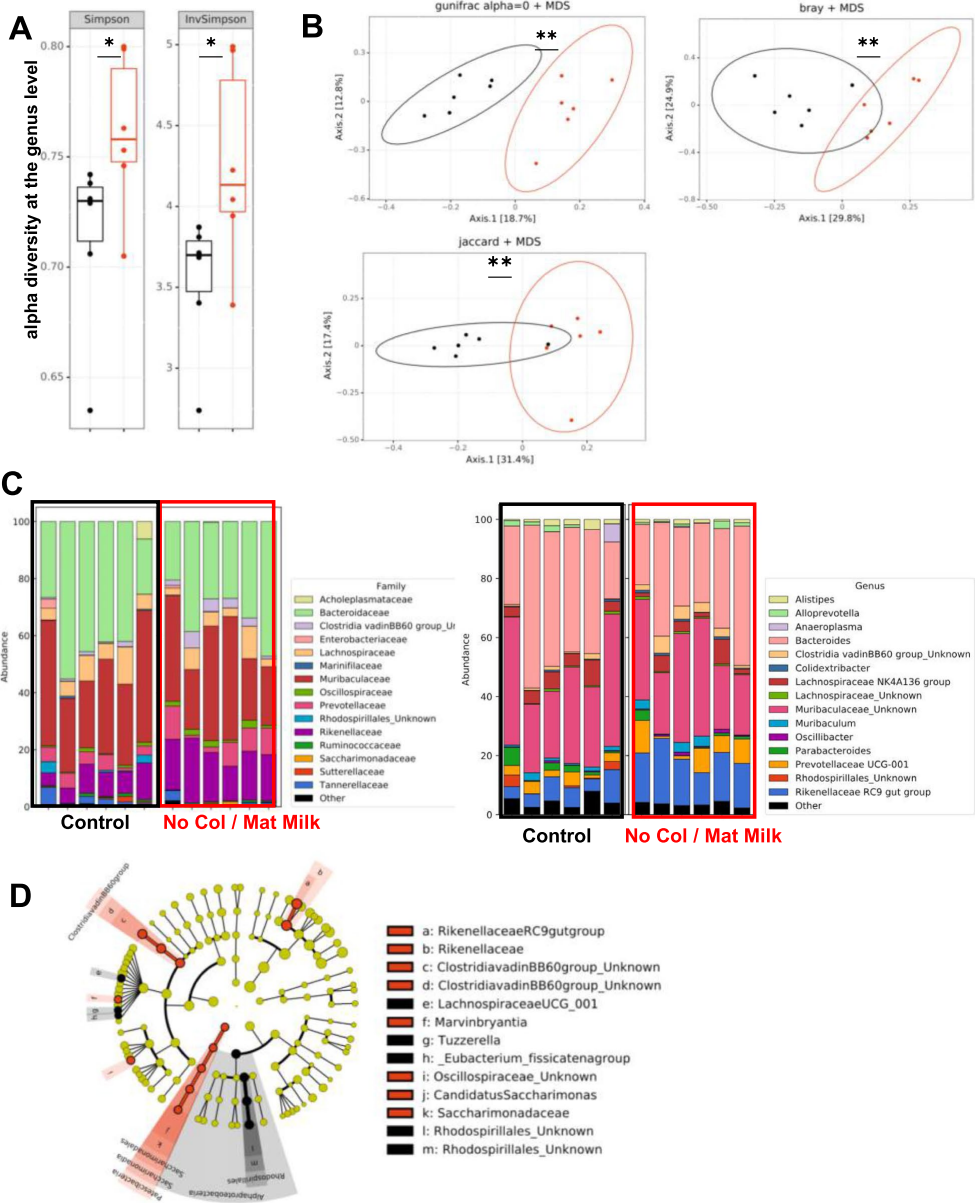
Colostrum shapes infant mice microbiota. 16S rRNA gene analysis of microbiota in faeces from 20-day-old specific pathogen-free (SPF) mice reared with (black) and without colostrum (red). **A** Alpha diversity (Simpson, InvSimpson) at the genus level; and (**B**) Beta diversity (using GUniFrac a = 0, Bray–Curtis and Jaccard distances) at the operational taxonomic units (OTU) level. **C** relative abundance bar plot of the 15 most abundant taxa at the family (left panel) and genus (right panel) taxonomic levels (**D**) LEfSe cladogram with log(LDA) score threshold of 2. Statistical analysis was performed using Wilcoxon-Mann–Whitney (alpha diversity) and Permanova (beta diversity) tests on 1 experiment with *n* = 6 per group. *P < 0.05, **P < 0.01

Given the recent publications stressing the importance of the cross-talk between the diet and the gut microbiota for healthy growth and a functional GH-IGF-1 axis [2, 6–9], we then evaluated whether the gut microbiota played a causal role in the failure of postnatal ignition of the somatotropic axis in No Colostrum mice. We first analysed the 16S rRNA gene sequences from the gut microbiota of Control and No Colostrum mice at 2 weeks of age. The sequencing effort for all samples was sufficient to obtain an exhaustive coverage of the bacterial community profiles present in the samples (Fig. S4). The average number of raw read pairs was 56,340 and the average number of read pairs classified in OTU was 35,964. The alpha diversity at the genus level of the Simpson and InvSimpson indexes showed significant differences between the two groups (Fig. 4A), indicating the microbiota evenness in the colostrum-deprived group was higher than the control one. The Beta-diversity was statistically different between groups using various distance parameters (GUniFrac α = 0, *p* = 0.002; Bray–Curtis *p* = 0.002; Jaccard *p* = 0.003) (Fig. 4B), showing that colostrum deprivation at birth induced a major change of the gut microbiota ecology during suckling. Finally, the bar plot analysis of the distribution of the taxa identified at the family and genus taxonomic level (Fig. 4 C) and the Linear discriminant analysis Effect Size (LEfSe) [27] showed significant differences in the distribution of the taxa between the 2 groups (Fig. 4 D), further reinforcing the impact of colostrum deprivation on microbial ecology.

**Fig. 5:**
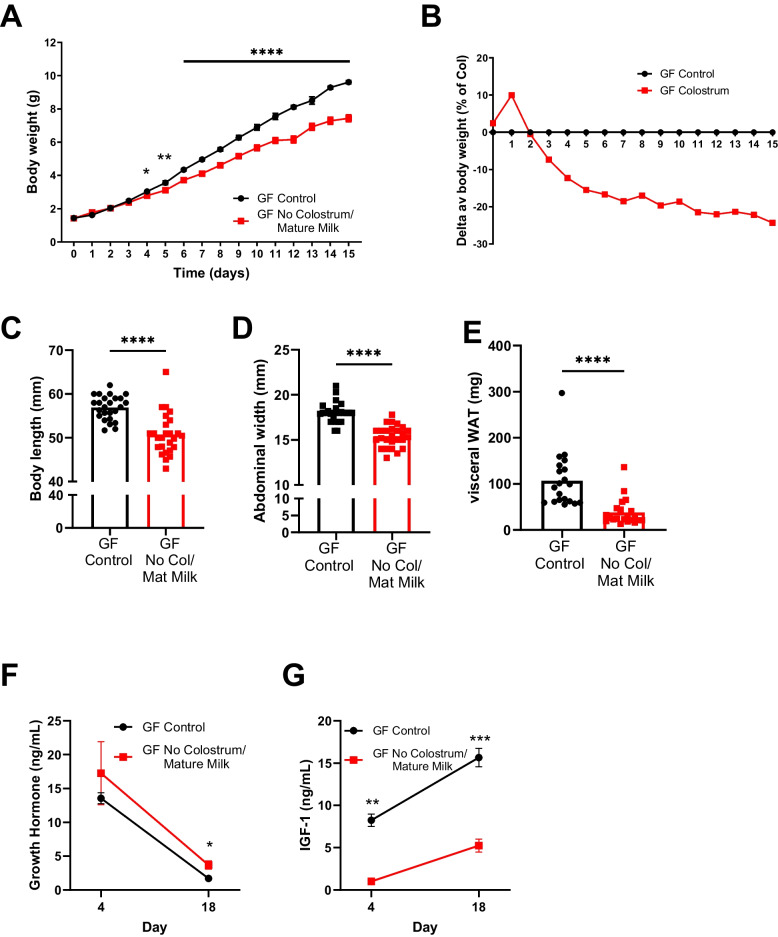
Growth retardation in colostrum-deprived mice is microbiota-independent. Colostrum deprivation in germ-free (GF) mice (**E**) Body weight curve of GF mice before weaning and (**F**) body weight as a percentage of the GF control group in GF Control (black) and GF No Colostrum (red) mice (**G**) Body length (**H**) Abdominal width (**I**) Visceral WAT weight (**J**) Growth hormone and (**K**) IGF-1 in plasma of GF mice. Data are presented as means with individual values. 3 experiments with *n* = 4–12 per group (**E**, **F**, **G**, **H**), 2 experiments with *n* = 9–12 per group (**I**), 1 experiment with *n* = 4–8 (**J**) and *n* = 5–8 (**K**) per group. Statistical analysis was performed using Mann–Whitney test. *P < 0.05, **P < 0.01, ***P < 0.001, ****P < 0.0001

To determine if the differences in gut microbiota diversity and composition in colostrum-deprived/mature milk fed mice are causally related to growth failure, we assessed the growth parameters of germ-free (GF) mice reared with or without colostrum. GF mice deprived of colostrum demonstrated growth retardation, as shown by their reduced body weight (Fig. 5A,B), body length (Fig. 5C), abdominal width (Fig. 5D) as well as visceral WAT weight (Fig. 5E) compared to control GF mice. Growth failure reached a decrease of 25% compared to Control mice, similar to what we observed in colostrum-deprived SPF mice (Fig. 1D), as well as in colostrum-deprived mice nursed by GF dams colonized by faecal microbial transplant (FMT) (Fig. S5A-B). Furthermore, colostrum-deprived GF mice also showed a state of GH resistance. Plasma GH levels were similar or increased in the GF No Colostrum group (Fig. 4J), while IGF-1 was significantly reduced (Fig. 5F). This was also observed in the GF mice colonized with FMT (Fig. S5F-G).

Altogether these data show that, even though colostrum shapes the composition of the establishing gut microbiota, the crosstalk between the newborn’s physiological diet and the microbiota is not required for colostrum promotion of growth. Recent observations have shown that microbial communities from undernourished children were sufficient to transfer growth failure in gnotobiotic mice at weaning [2]. To evaluate whether the microbiota of growth retarded colostrum-deprived mice could also negatively impact growth, Control and No Colostrum pups were cohoused with GF mice at weaning. Cohousing of GF mice with No Colostrum pups resulted in growth outcomes that were similar to GF mice cohoused with Control mice (Fig. S6A-B), which further supports that the microbiota is not playing a causal role in the growth failure of colostrum-deprived mice.

## Discussion

This study establishes a preclinical model of stunted growth due to growth hormone resistance, despite mice being exclusively breastfed by healthy mothers, raised in the absence of pathogens, and persisting in the absence of microbiota. Specifically, this study showed that feeding mature milk rather than colostrum from birth has a dramatic negative effect on successful growth, further emphasising the importance of dynamic changes in the composition of breast milk to match the developmental stages of neonates, as previously shown in marsupials [28]. While many studies have demonstrated the critical role of breastmilk in shaping the gut microbiota [29, 30], none have specifically addressed the importance of the lactation stage. Here, we showed that colostrum feeding at birth is essential in determining gut microbiota ecology. However, while there is growing literature on the role of microbiota in promoting healthy growth after weaning [2], we unexpectedly found that growth benefits associated with colostrum feeding do not require microbiota.

The GH–IGF-1 axis is a key endocrine mechanism regulating linear growth in children [24, 25]. Many of the actions of GH are mediated by IGF-1 and include anabolic effects on bone. One of the main causes of acquired growth failure is GH resistance, where IGF-I levels are low despite normal or high levels of GH. A major deficit in nutrient intake or infection, which are the major causes of acquired GH resistance [24, 31, 32], could not explain GH resistance in colostrum-deprived mice. We further tested the role of compounds that are high in colostrum and know to play a role in the regulation such as vitamin A, zinc, EGF, TGF-beta and none of these factors could rescue growth in colostrum-deprived mice and/or their neutralisation in the control group resulted in growth failure (data not shown). Recently, Schwartzer et al. and Yan et al. demonstrated that the microbiota was key in enabling the somatotropic axis and driving growth in juvenile and adult mice [6, 7]. The importance of bacterial NOD2 ligands was further shown to be key in promoting post weaning growth [9]. The need for crosstalk between the diet and the gut microbiota for the prevention of growth failure was also shown in elegant translational studies as recently reviewed by Barratt et al. [2]. Our study demarks from these pioneering findings as we found that the growth-promoting effect of colostrum was independent of the microbiota. Interestingly, the need for crosstalk between the diet and the gut microbiota for the prevention of growth failure had only been studied after weaning [2, 6–8] while our study addresses its role before weaning. At this stage of development, a stable microbiota has not been established yet [30], which may explain the smaller role of the microbiota at the early stage and underly the direct effects of the early diet on the somatotropic axis. We may further hypothesise that colostrum contains endogenous ligands that are similar to Lactiplantibacillus plantarum NOD2 ligands and were demonstrated to be required for post-weaning growth [9]. A limitation to the interpretation and potential translatability of our data to the human setting is the difference in HMOs content, which are in low abundance and variety in mouse milk compared to human milk [33]. However, despite this difference, colostrum feeding shaped the microbiota and played a role in growth in early life in our mouse model. Therefore, our data highlight other physiological pathways in the regulation of growth by early nutrition that may be conserved across species. Importantly, while the majority of stunting interventions have been targeted at improving complementary feeding practices after 6 months of age, accumulating data shows an already high prevalence of stunting before 6 months of age [3]. Strikingly, our data point towards the importance of early initiation and exclusive breastfeeding during the first days of life for growth promotion. Only very few studies have addressed this question in clinical trials and their data support our findings [35].

The preclinical model investigated here may be extremely relevant in the context of growth failure in preterm infants. Two recent clinical studies found improved growth outcomes in very low birth weight infants fed their mother’s milk, compared with donor human milk (which consists of pooled milk collected at advanced stage of lactation) [36–38]. Our observations support the hypothesis that the mismatch between the lactation stage and newborn’s age may play an important role in retarded growth in newborns fed (mature) donor human milk instead of mother’s own milk [28, 39].

A limitation of our study is that in the model we used, we cannot disentangle the benefits of colostrum from the detrimental effects of mature milk at birth. We expect that our data will stimulate further research to identify which factors in colostrum promote growth and/or which factors in mature milk are responsible for growth failure, and open up new avenues for promoting healthy growth in vulnerable newborns through age-appropriate nutritional intervention.

## Conclusion

This study reveals the importance of diet at birth for the imprinting of healthy growth and development through a functional somatotropic axis, independently of the microbiota. We expect our work will foster novel paths of translational research leading to the identification of bioactives that are required for optimal newborn growth and will provide the scientific evidence required for the efficient promotion of colostrum feeding. These are promising new avenues for decreasing the burden of child undernutrition.

## Methods

### Materials & methods

#### Mice

Time-mated pregnant Balb/c dams (Animal Research Centre, Canning Vale, Australia) were housed in pairs in individually ventilated cages at the Harry Perkins Institute for Medical Research. Gnotobiotic (germ-free) BALB/c dams were sourced from the Translational Research Institute (Queensland, Australia) and housed in positively pressurised, high-efficiency particulate air (HEPA) filtered isolators, at the SAHMRI Preclinical, Imaging and Research Laboratories. Germ-free status was confirmed upon arrival and each time the isolator was opened by collecting swabs and faecal samples for culture under aerobic and anaerobic conditions (Compath monitoring services) as well as via 16S rRNA gene qPCR of DNA extracted from faecal samples [40]. All mice had access to autoclaved commercially pelleted food and sterilised water ad libitum with regulated daylight, humidity, and temperature. All experimental procedures used were approved by the Harry Perkins Institute for Medical Research or SAHMRI Animal Ethics Committee and performed according to institutional guidelines.

### Colostrum fostering model

Time-mated pregnant dams were monitored hourly around birth. Newborn pups were removed from the dams within one hour after birth, before the first feed. Newborn pups were randomly distributed and fostered to a pair of dams that either just had given birth (colostrum feeding) or to a pair of dams that gave birth nine days earlier (No Colostrum feeding) (Fig. 1A). Body weight and other morphometric measurements were recorded over the course of the experiment. Crown-to-rump length was measured from the highest point of the head to the lowest point of the rump. Pups were culled using a lethal injection with pentobarbital.

### Micro-CT imaging adipose tissue

Micro-CT imaging was performed using a Skyscan 1176 MicroCT (Bruker, Kontich, Belgium) on 2-week-old pups anaesthetized with isoflurane. Isoflurane was maintained at 2.5% mixed with 0.8L/min Oxygen. Acquisition settings were as following: Voltage 50 kV, Exposure 92 ms, Current 500 μA, Filter Al 0.5 mm thick, Pixel size 35.55 μm isotropic, Rotation step 0.7°, Frames averaging of 2 and a 360˚ scan. Data was reconstructed using the NRecon software (V1.7.1.0) with the following settings: Smoothing 3, Ring Artifact Correction 8, Beam Hardening Correction 10%, Thresholding 0- 0.02 X-Ray attenuation coefficients and Defect Pixel Masking 3%.

CT Analysis involving quantification of white adipose tissue (WAT) and brown adipose tissue (BAT) was done using Bruker CTAn software V1.20.8.0. For every dataset, the top reference slice was the base of the tail, and the bottom reference slice was the start of the neck or separation of the Anterior Cervical and the Supraclavicular BAT or start of Classical BAT. As the density of lungs is like that of adipose tissue in CT datasets, before proceeding with the automated WAT and BAT analysis, it was important to create Regions of Interests (ROI) encompassing the lungs (Start- branching of the trachea into bronchi; End- disappearance of the lungs), so that these could be eliminated during automated analysis as part of the workflow.

The Lean Body Mass (LBM) was calculated by subtracting the total adipose tissue mass from the body weight. The adipose tissue mass was calculated using its density of 0.95 kg/L.

### Bone measurements and micro-CT bone analysis

The lengths of the dissected leg bones (femurs) were measured using digital callipers. Bones were fixed 4% of paraformaldehyde-PBS overnight at 4 °C and then stored in 70% ethanol. The leg bones were then removed from PBS, wrapped in a PBS-soaked Kim wipe, and placed in an airtight 5 ml plastic bottle and scanned individually using the Skyscan 1176 MicroCT (Bruker, Kontich, Belgium). Acquisition settings were as following: Voltage 40 kV, Exposure 1020 ms, Current 600 μA, no filter used, Pixel size 9 μm isotropic, Rotation step 0.3°, Frames averaging of 2 and a 360˚ scan. Data was reconstructed using the NRecon software (V1.7.4.6) with the following settings: Smoothing 1, Ring Artifact Correction 8, Beam Hardening Correction 30%, Threshold values 0.00–0.15 X-Ray attenuation coefficients. Bruker’s CTAn software (V1.20.8.0, Bruker, Kontich, Belgium) was used for bone analysis. For trabecular bone analysis the top-end slice for the region of interest was identified at 0.5 mm (offset) distally from the growth plate, towards the diaphysis and the bottom-end slice was defined at 0.5 mm (height) from the top-end slice. For cortical bone measurements, the offset was 1.74 mm from the growth plate and 1 mm was used as the height for the region of interest to measure 2D and 3D parameters.

### Adipose tissue histology

Perigonadal white adipose tissue was fixed in 4% paraformaldehyde-PBS overnight at 4 °C and then stored in 70% ethanol. Automated overnight processing of the tissues was performed in a Leika ASP 200 Tissue Processor (Germany), after which the tissues were embedded in paraffin pax (Lab Serv, USA). Paraffin Sects. (5 μm) were fixed on a Superfrost™ slide (ThermoFisher, Germany), dewaxed and stained with Hematoxylin (Lab Supply, Australia) for 1.5 min and Eosin (Proscitech, Australia) for 45 s. The slides were mounted with a coverslip with dibutylphthalate polystyrene xylene (DPX; Leica, USA). Five high power fields were captured for three slices per sample at 400 × magnification. The adipocyte size was determined manually by tracing the adipocyte perimeter using NIS Element software as described [41]. The border of individual adipocytes was traced using the Bezier Region of Interest tool. 20 Adipocytes per HPF were measured, ensuring a total of 300 adipocytes per sample. To ensure randomisation, the adipocyte in the bottom left hand was traced first, followed by the adipocyte directly above it. Adipocytes touching the border of the image were excluded from analysis.

### White adipose tissue immune cell isolation

Perigonadal and retroperitoneal fats were weighed, combined and cut into a pulp in DPBS/0.5% BSA and digested with 1 mg/ml collagenase type II from Clostridium histolyticum (Sigma Aldrich C6885) in DPBS with 0.5% BSA and 10 mM CaCl2 (Sigma) for 10 min at 37 °C on a shaker. EDTA was added to stop the reaction and the suspension was filtered through a 70 µm cell strainer (Falcon, BD), followed by a 40 µm cell strainer and spun at 140 xg for 15 min to pellet cells.

### Flow cytometry

For staining, WAT cells (up to 5 × 10^6 cells per well) were plated in 96 well round bottom tissue culture plates (Costar, Corning, USA) and stained for 30 min with fixable viability stain in PBS to exclude dead cells (LIVE/DEAD Fixable Near-IR Dead Cell Stain kit, Invitrogen, Thermo Fisher Scientific, MD, USA). After washing, the cells were incubated with the relevant antibody cocktail including 2.4G2 to block Fc-receptors, in 100 μl FACS buffer (RMPI with 10% FCS and 2 mM EDTA) for 30 min at 4 °C. The following anti-mouse antibodies from BD Biosciences, unless otherwise stated, were used: CD45-PE (30-F11), CD4-BV650 (RM4-5), CD3-FITC (17A2)(Biolegend, CA, USA), B220-FITC (RA3-6B2)(Biolegend), CD11b-FITC (M1/70), CD90.2-eFluor450 (53–2.1)(Invitrogen, Life Technologies, CA, USA). Cells were either fixed in 0.1% paraformaldehyde overnight or fixed using the eBioscience FoxP3/Transcription Factor Staining Buffer set (San Diego, CA, USA), according to the manufacturer’s instructions, prior to addition of intracellular stains from Invitrogen eBioscience (FoxP3-PE-Cy7 (FJK-16 s), GATA3-PerCp-eFluor710 (TWAJ), ROR gamma (t)-APC (B2D)) for 30 min. Samples were acquired on a 5-laser BD LSRFortessa flow cytometer using FACSDiva software (BD Biosciences, New Jersey, USA) and results analyzed using the FlowJo v10.8 software (Tree Star, Ashland, USA). Fluorescence spill over was compensated in FACSDiva software using single-stained controls, and manually adjusted in FlowJo for data analysis.

### Blood markers

Cardiac puncture was used to collect blood in heparin tubes (Sigma). Plasma was analysed for cytokines using the High Sensitivity 5-Plex Mouse ProcartaPlex™ Panel (ThermoFisher). The mouse TNF-α uncoated ELISA kit (Invitrogen, ThermoFisher) was used with adaptations to the protocol to run the assay in 50ul per well. Growth hormone was measured by Rat/Mouse Growth Hormone ELISA Kit (Millipore). Insulin-like growth factor (IGF)-1 was measured by ELISA (Duoset ELISA; R&D Systems) with slight adaptations to the manufacturer’s protocol. In brief, all incubation volumes were halved to perform the assay in 25ul per well. Triglycerides (Trig2), cholesterol (Chol2), high-density lipoprotein (HDL) cholesterol (Ultra HDL) and low-density lipoprotein (LDL) cholesterol (Direct LDL) were quantified on the Alinity c system (Abbott, IL, USA) by PathWest Laboratory Medicine WA (Perth, WA, Australia). In addition, the Triglyceride Colorimetric Assay Kit from (Cayman Chemical, MI, USA) was used. BioRad Bioplex Pro Mouse Diabetes 8-plex was used to determine leptin concentrations.

### 16S rRNA Gene sequencing

The data presented in the study are deposited in the ENA repository, accession number PRJEB59835. DNA was extracted from faeces samples using the QIAamp DNA Stool kit (Qiagen, Hilden, Germany) as described previously [42]. The sequencing was performed using the Illumina® MiSeq technology after a two-step PCR library preparation [42]. Briefly, The V3-V4 hyper-variable regions of the 16S rRNA gene were amplified from the DNA extracts during a first PCR step using universal 16S primers (Vaiomer, Toulouse-Labège, France). The expected amplicon lengths were between 350 and 500 base pairs (bp). For each sample, a sequencing library was generated by addition of sequencing adapters and multiplexing indexes during a second PCR step before sequencing on an Illumina MiSeq machine in 2 × 300 bp paired-end reads mode. The targeted metagenomic sequences from microbiota were analyzed using the bioinformatics pipeline established by Vaiomer to find operational taxonomic units (OTUs) with Galaxy solution (FROGS v1.4.0) guidelines [43]. Briefly, after demultiplexing of barcoded Illumina paired reads, single-read sequences were cleaned, the last 10 and 50 bases of respectively, R1 and R2 reads were trimmed and paired into longer fragments. Amplicons shorter than 350 nt or longer than 500 nt were removed. OTUs were produced with single-linkage clustering in two passes of the FROGS embedded Swarm algorithm which uses aggregation distance instead of a fixed sequence identity clustering threshold: the first pass with an aggregation distance equal to 1 and the second pass with an aggregation distance equal to 3. OTUs with abundance lower than 0.005% of the whole dataset abundance were removed. The taxonomic assignment was performed by BLAST against SILVA 138.1 database to determine bacterial profiles from phylum to genus. All the samples passed the quality controls with more than 37,500 raw sequences and 5,000 quality-filtered sequences as illustrated on Fig S4. The overall median number of reads after filtering was 56,182 with 1st and 3rd quartiles being respectively 35,165.50 and 61,101.75 sequences.

### Statistics

The 16S rRNA amplicon gene sequencing graphics and statistical analyses were generated using custom Python scripts (Alpha and Beta diversities, ordinationsand taxonomic) with the following libraries: Scikit-Bio v0.4.2 for Alpha and Beta diversity calculations as well as Beta diversity ordinations, Scipy v1.2.1 for hierarchical clustering calculations as well as Kruskal–Wallis and Wilcoxon rank-sums tests on Alpha diversities, Plotnine v0.4.0 and MatPlotLib v2.2.4 for graphical representation of the results. A Wilcoxon-Mann–Whitney test was performed on the number of sequences classified in OTUs between the groups to ensure there was no statistically significant difference between the groups (U-value = 0.923, *p*-value = 0.337) before running the diversity analyses. Depending on the analyses, count data (alpha and beta diversities) or Total Sum Scaling (relative abundances) normalized data (taxa composition and LEfSe) have been used.

For other data than 16S rRNA amplicon gene sequencing, data were analysed with Mann–Whitney, using GraphPad Prism Software version 9.3.1 (La Jolla, CA, USA). Values of *P* < 0.05 were considered statistically significant.

A summary of all the experiments performed and the outcomes assessed in each of them is available in Supplementary Table 1.

### Supplementary Information

Supplementary file 1. Fig. S1. Phenotype at 2 weeks of age in mice reared with and without colostrum. (A) Lactation stage in mice. Pictures and content in macronutrients in mouse milk collected at various time points. (B) Representative microCT bone images; percentages (C) visceral and (D) subcutaneous (sc) WAT. Plasma (E) cholesterol (F) high-density-lipoprotein (HDL) cholesterol. Data are presented as means ± SEM. 1 experiment with 5-12 milk collected per experiment (A), 1 experiment with n=6-8 per group (C, D) and 3 experiments with n=3-5 per group (E, F) Statistical analysis was performed using Mann-Whitney test. *P < 0.05, **P < 0.01

Supplementary file 2. Fig. S2. Growth post weaning in mice reared with and without colostrum. (A) Body weight from 2 weeks onwards into adulthood (*n*=6-12 per time point). (B) Body length and (C) visceral white adipose tissue (WAT) weight at 7-week of age. Data are presented as means with individual values depicted or means ± SEM. Data from one experiment with *n* = 6-12 (A) or *n*=6 (B, C) per group. Statistical analysis was performed using Mann-Whitney test. **P < 0.01, ****P < 0.0001.

Supplementary file 3. Fig. S3. Milk consumption and gut absorptive capacity. (A) Milk consumption over a 2-hour time window (1 experiment with *n*=6/group day 1; 2 experiments with *n*=5-6 day 4 and 14). (B) Length of the small intestine (1 experiment *n*=6/group day 4, 2 experiments *n*=5-6 day 14) (C) Villus length (1 experiment *n*=4-5/group at day 4; 2 experiments *n*=3-6 at day 19) in the jejunum, with representative images of hematoxylin and eosin stained jejunum at day 4. (D) Colon content weight and percentage lipid in dry faeces (One experiment with *n*=6 per group, with *n*=3 pools of 2 samples for lipid content No Colostrum group). Data are presented as means ± SEM. Statistical analysis was performed using Mann-Whitney test. *P < 0.05, **P < 0.01, ***P < 0.001, ****P < 0.0001

Supplementary file 4. Fig. S4. Read counts. Number of raw read pairs (brown) and read pairs that were classified into OTU (blue) per sample. The red line illustrates the targeted 37,500 raw read pairs per sample, which was empirically determined to be the number of read pairs to obtain an exhaustive coverage of the bacterial community profiles present in high diversity samples. The green line at 5,000 read pairs depicts the required minimum number of read pairs classified in OTUs. 1 experiment with *n*=6/group.

Supplementary file 5. Figure S5. Growth and metabolic parameters in germ-free mice colonised using faecal microbial transplant (FMT) and reared with and without colostrum. (A) Body weight growth curve before weaning and (B) body weight as a percentage of the FMT control group (*n*=10 per group). (C) Body length and (D) abdominal width. FMT pups were culled at day 20 to determine (E) Visceral white adipose tissue (WAT) weight (F) growth hormone and (G) insulin-like growth factor-1 (IGF-1). Data are presented as means with individual values depicted or means ± SEM. Data from 1 experiment with *n*=10/group (A-E) or *n*=7-8/group (F, G). Statistical analysis was performed using Mann-Whitney test. *P < 0.05, **P < 0.01, ***P < 0.001, ****P < 0.0001

Supplementary file 6. Figure S6. Microbiota from pups reared with and without colostrum have a similar capacity to promote growth after weaning. (A) Body weight growth curve of germ-free (GF) mice (no cohousing) and GF mice cohoused at weaning with mice reared with (GFxControl) or without (GFxNo colostrum) (*n*=6 per group). (B) Visceral white adipose tissue (WAT) weight after four weeks of cohousing. Data are presented as means with individual values depicted or means ± SEM. Data from one experiment with *n*=6/group. Statistical analysis was performed using Mann-Whitney test. *P < 0.05, **P < 0.01, ***P < 0.001, ****P < 0.0001

Supplementary file 7. Supplementary Table 1. Experiments design.

## Data Availability

The 16S rRNA gene sequencing data presented in the study are deposited in the ENA repository, accession number PRJEB59835. All the other are available in the main text, on GitHub (https://github.com/vaiomer/Van_Den_Elsen_et_al), or the supplementary materials.
