# Supplementary figures and images for: Diet at birth is critical for healthy growth, independent of effects on the gut microbiota
Source: Microbiome. 2024 Jul 27;12:139. doi: 10.1186/s40168-024-01852-7 (PMC11282663; doi:10.1186/s40168-024-01852-7)

**A**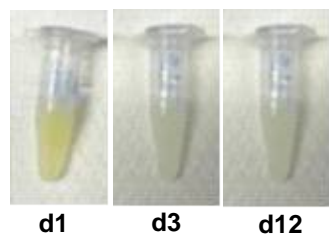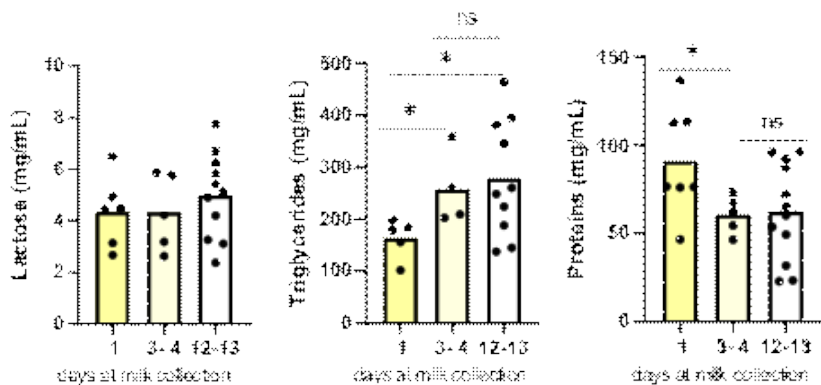**B**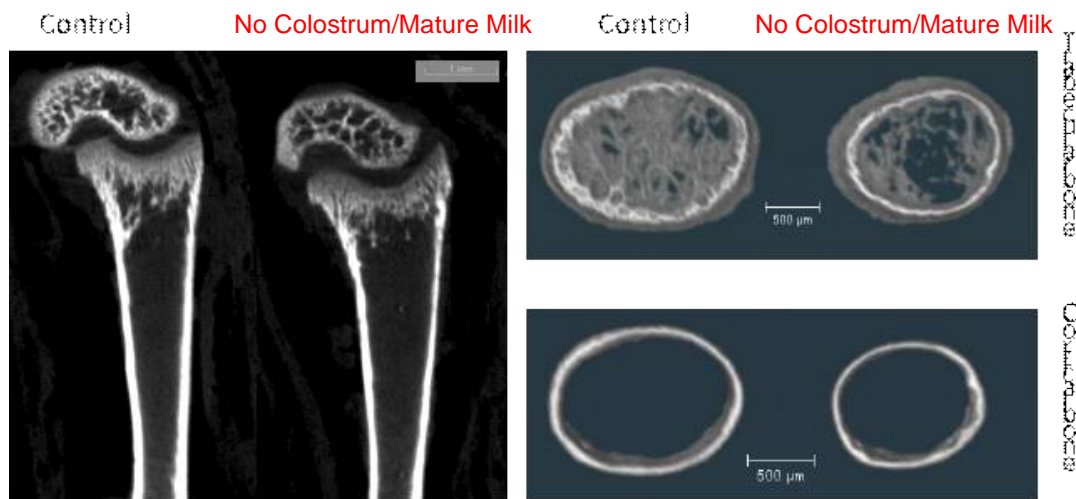**C**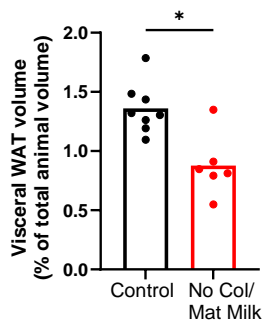**D**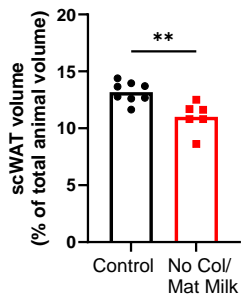**E**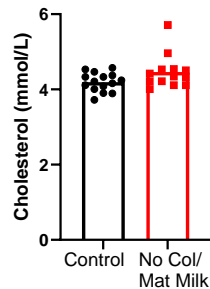**F**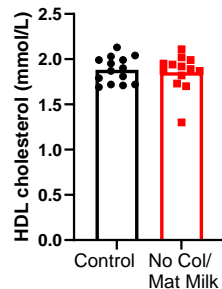

Supplement: Supplementary file 2 — Supplementary file 1. Fig. S1. Phenotype at 2 weeks of age in mice reared with and without colostrum. (A) Lactation stage in mice. Pictures and content in macronutrients in mouse milk collected at various time points. (B) Representative microCT bone images; percentages (C) visceral and (D) subcutaneous (sc) WAT. Plasma (E) cholesterol (F) high-density-lipoprotein (HDL) cholesterol. Data are presented as means ± SEM. 1 experiment with 5-12 milk collected per experiment (A), 1 experiment with n=6-8 per group (C, D) and 3 experiments with n=3-5 per group (E, F) Statistical analysis was performed using Mann-Whitney test. *P < 0.05, **P < 0.01 [file 40168_2024_1852_MOESM1_ESM.pdf]

**A**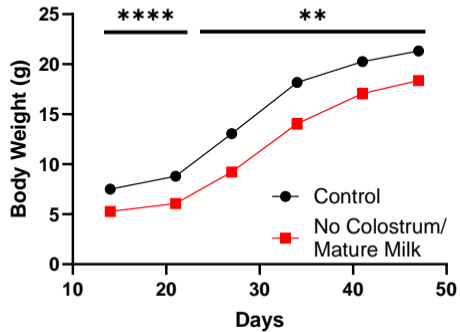**B**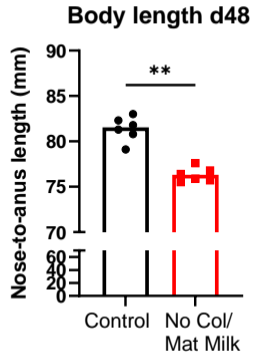**C**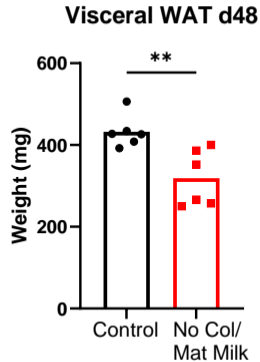

Supplement: Supplementary file 3 — Supplementary file 2. Fig. S2. Growth post weaning in mice reared with and without colostrum. (A) Body weight from 2 weeks onwards into adulthood (n=6-12 per time point). (B) Body length and (C) visceral white adipose tissue (WAT) weight at 7-week of age. Data are presented as means with individual values depicted or means ± SEM. Data from one experiment with n = 6-12 (A) or n=6 (B, C) per group. Statistical analysis was performed using Mann-Whitney test. **P < 0.01, ****P < 0.0001. [file 40168_2024_1852_MOESM2_ESM.pdf]

**A**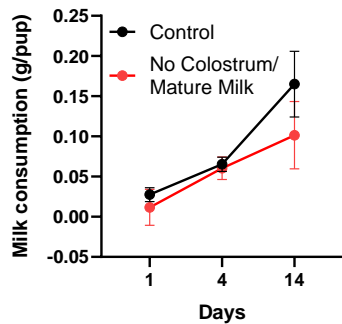**B**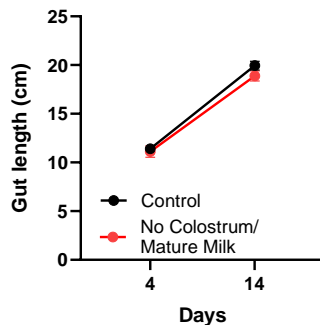**D**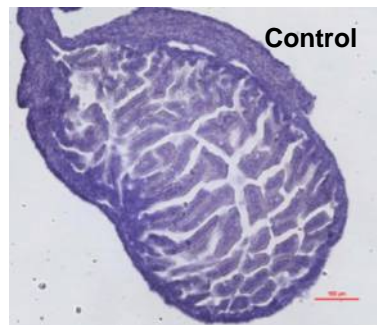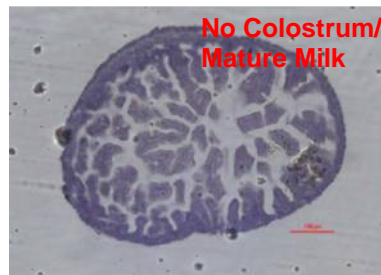**C**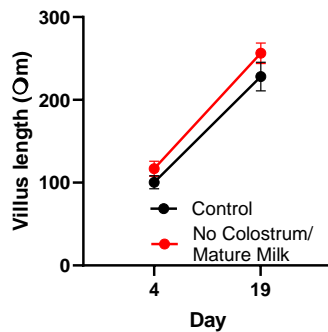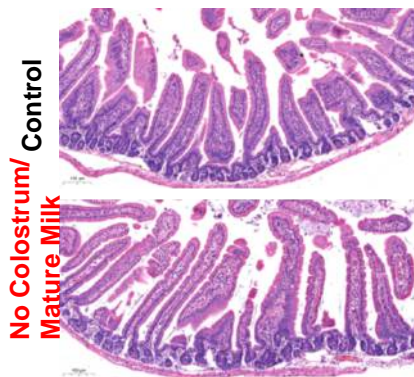**E**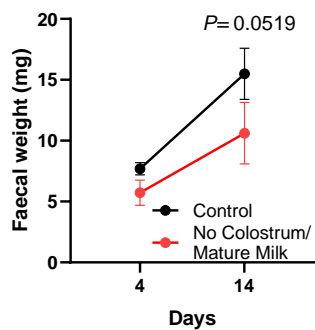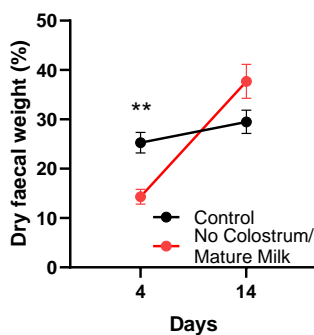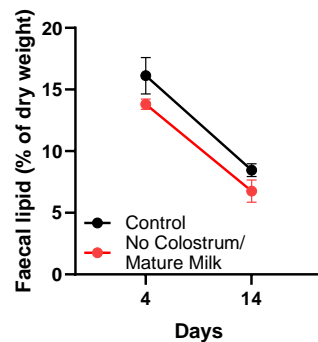

Supplement: Supplementary file 4 — Supplementary file 3. Fig. S3. Milk consumption and gut absorptive capacity. (A) Milk consumption over a 2-hour time window (1 experiment with n=6/group day 1; 2 experiments with n=5-6 day 4 and 14). (B) Length of the small intestine (1 experiment n=6/group day 4, 2 experiments n=5-6 day 14) (C) Villus length (1 experiment n=4-5/group at day 4; 2 experiments n=3-6 at day 19) in the jejunum, with representative images of hematoxylin and eosin stained jejunum at day 4. (D) Colon content weight and percentage lipid in dry faeces (One experiment with n=6 per group, with n=3 pools of 2 samples for lipid content No Colostrum group). Data are presented as means ± SEM. Statistical analysis was performed using Mann-Whitney test. *P < 0.05, **P < 0.01, ***P < 0.001, ****P < 0.0001 [file 40168_2024_1852_MOESM3_ESM.pdf]

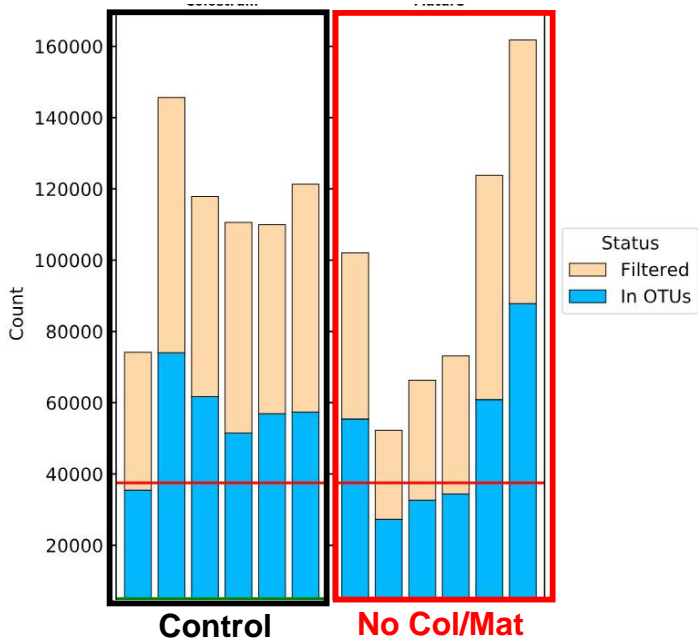

Supplement: Supplementary file 5 — Supplementary file 4. Fig. S4. Read counts. Number of raw read pairs (brown) and read pairs that were classified into OTU (blue) per sample. The red line illustrates the targeted 37,500 raw read pairs per sample, which was empirically determined to be the number of read pairs to obtain an exhaustive coverage of the bacterial community profiles present in high diversity samples. The green line at 5,000 read pairs depicts the required minimum number of read pairs classified in OTUs. 1 experiment with n=6/group. [file 40168_2024_1852_MOESM4_ESM.pdf]

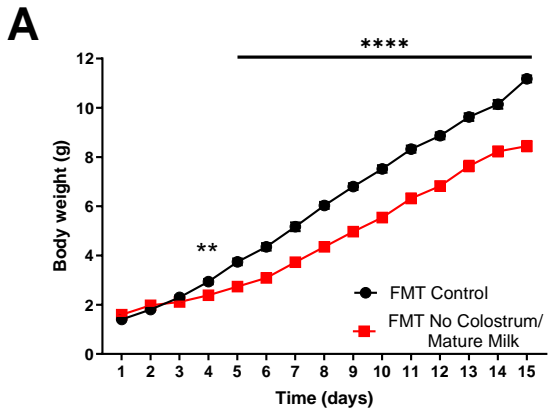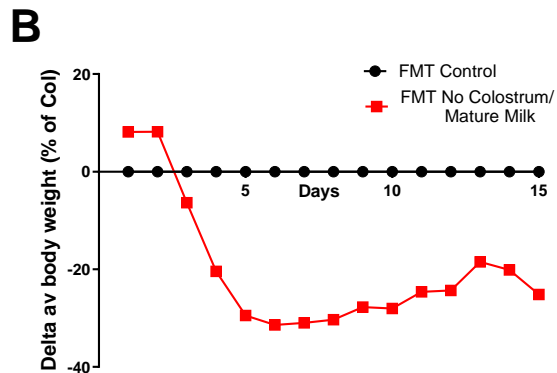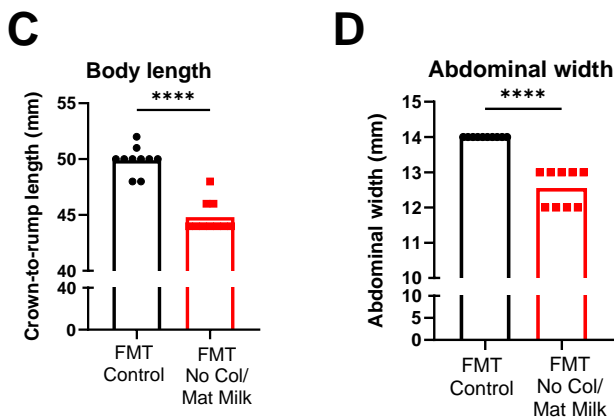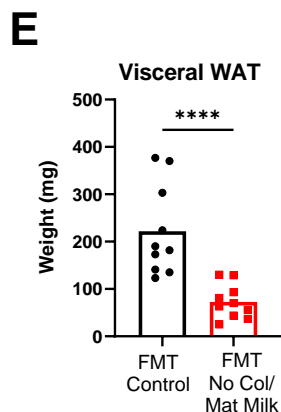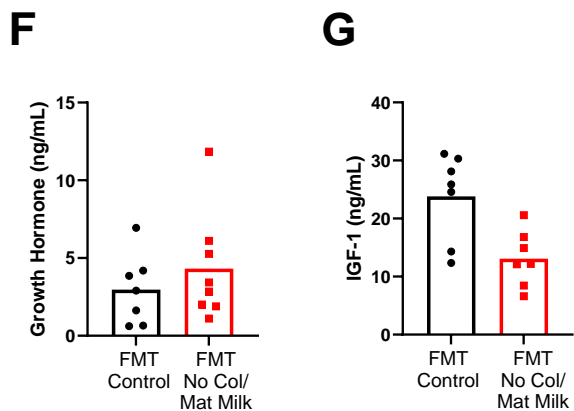

Supplement: Supplementary file 6 — Supplementary file 5. Figure S5. Growth and metabolic parameters in germ-free mice colonised using faecal microbial transplant (FMT) and reared with and without colostrum. (A) Body weight growth curve before weaning and (B) body weight as a percentage of the FMT control group (n=10 per group). (C) Body length and (D) abdominal width. FMT pups were culled at day 20 to determine (E) Visceral white adipose tissue (WAT) weight (F) growth hormone and (G) insulin-like growth factor-1 (IGF-1). Data are presented as means with individual values depicted or means ± SEM. Data from 1 experiment with n=10/group (A-E) or n=7-8/group (F, G). Statistical analysis was performed using Mann-Whitney test. *P < 0.05, **P < 0.01, ***P < 0.001, ****P < 0.0001 [file 40168_2024_1852_MOESM5_ESM.pdf]

**A**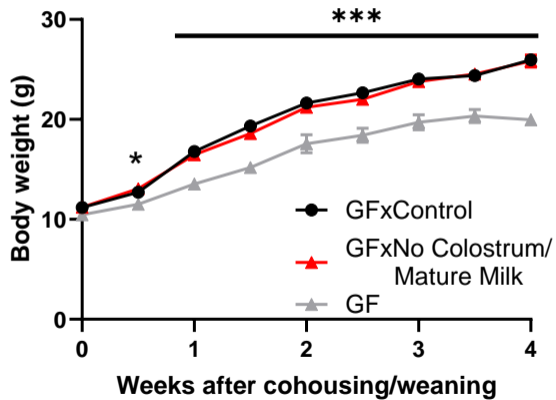**B**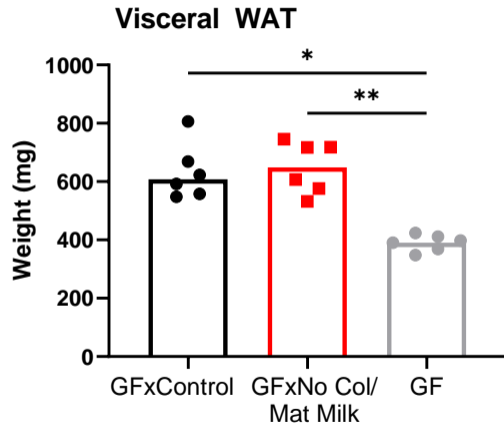

Supplement: Supplementary file 7 — Supplementary file 6. Figure S6. Microbiota from pups reared with and without colostrum have a similar capacity to promote growth after weaning. (A) Body weight growth curve of germ-free (GF) mice (no cohousing) and GF mice cohoused at weaning with mice reared with (GFxControl) or without (GFxNo colostrum) (n=6 per group). (B) Visceral white adipose tissue (WAT) weight after four weeks of cohousing. Data are presented as means with individual values depicted or means ± SEM. Data from one experiment with n=6/group. Statistical analysis was performed using Mann-Whitney test. *P < 0.05, **P < 0.01, ***P < 0.001, ****P < 0.0001 [file 40168_2024_1852_MOESM6_ESM.pdf]
