# Supplementary material for: Diet at birth is critical for healthy growth, independent of effects on the gut microbiota
Source: Microbiome. 2024 Jul 27;12:139. doi: 10.1186/s40168-024-01852-7 (PMC11282663; doi:10.1186/s40168-024-01852-7)
Supplement: Supplementary file 8 — Supplementary file 7. Supplementary Table 1. Experiments design. [file 40168_2024_1852_MOESM7_ESM.pdf]

Supplementary Table 1. Experiments' design

SPF												
**Parameter**	**Body weight**	**Body length**	**Body width**	**uCT**	**WAT weight**	**adipocyte size**	**WAT flow**	**Blood lipids**	**Leptin/cytokines**	**GH/IGF day 4**	**GH/IGF day 7**	**GH/IGF day 15**	**16S**
**Figure**	**1A-C**	**1C**	**1D**	**1H-K, 2A-B**	**2D**	**2F**	**2G-H**	**2I-J**	**2K-M**	**3**	**3**	**3**	**4**
**n= expt**	**5**	**4**	**3**	**1**	**3**	**2**	**3**	**3**	**4**	**1**	**2**	**4**	**1**
**Expt 1**	10 / 10	9 / 5											6 / 6
**2**	8 / 6	8 / 6	8 / 6	8 / 6		8 / 6							
**3**	10 / 9	10 / 8	10 / 8		10 / 8		8 / 8	5 / 5	5 / 5			7 / 7	
**4**	14 / 14				7 / 6		7 / 6	5 / 5	5 / 5		5 /5	5 / 5	
**5**	12 / 12	12 / 12	12 / 12										
**6**						6 / 5							
**7**					5 / 6				6 / 6			5 / 5	
**8**								4 / 3	8 / 6	8 / 8		5 / 5	
**9**							1 /1 pooled				5 /5		
**Note**	Effect observed in every experiment, weight measured as frequently (daily) after expt 5 therefor not included	Not measured in other expt to reduce handling	Not measured in other expt to reduce handling	N=1 due to cost / Fig1K n=2-3 trabecular bones damaged in processing		Not measured in other expt, WAT used for different parameters	max n=8/group for technical limitations (processing times)	Blood volume limited, utilized for other measurements too	Blood volume limited, utilized for other measurements too	n=1 due to large plasma volume required & endpoint day 4	N=2 due to large plasma volume required & endpoint 7	Blood volume limited, utilized for other measurements too	N=1 due to cost
							Expt 9 cells pooled due to low cell yield						

Germ-free				
**Parameter**	**GF weight/length**	**GF WAT**	**GF GH/IGF1 day**	**F GH/IGF1 day 15**
**Figure**	**5A-D**	**5E**	**5E**	**5E**
**n= expt**	**3**	**2**	**1**	**1**
**Expt GF1**	4 / 5			
**GF2**	11 / 12	11 / 12		
**GF3 FMT**	10 / 9	10 / 9	5 / 5	8 / 8
**Note**		WAT not collected in GF1	blood volume limited	blood volume limited

Numbers indicate number of mice for the control group (left) and no colostrum/mature milk group (right)

Supplements																
**Parameter**	**Milk**	**uCT**	**Cholesterol**	**Weight 6wks**	**length/WAT d48**	**consumption d**	**consumption day**	**Gut length d4**	**Gut length d14**	**Villus length d4**	**Villus length d1**	**Faecal lipid**	**Read counts**	**Growth FMT**	**GH/IGF FMT**	**Cohousing**
**Figure**	**S1A**	**S1C-D**	**S1E-F**	**S2A**	**S2B-C**	**S3A**	**S3A**	**S3B**	**S3B**	**S3C**	**S3C**	**S3E**	**S4**	**S5A-E**	**S5F-G**	**S6**
**n= expt**	**2**	**1**	**3**	**1**	**1**	**1**	**1**	**1**	**2**	**1**	**2**	**1**	**1**	**1**	**1**	**1**
**Expt 1**													6 / 6			
**2**		8 / 6														
**3**			5 / 5													
**4**			5 / 5								3 / 4		5 / 5 (not shown)			
**5**				6-12 per timepoi	6 / 6			5 / 6	5 / 6		5 / 6					6 / 6
**6**																
**7**																
**8**			5 /3						6 / 5	5 / 4		6 / 6				
**9**	5 (day 14)															
**10**	7					6 / 6	11 / 11									
**GF3 FMT**														10 / 10	8 / 8	
**Note**	Milk volume limited, not enough to measure lactose, TG, protein in the same sample	n=1 due to cost	Blood volume limited	N=1 as a proof of principle, adult age outside scope of paper	N=1 as a proof of principle, adult age outside scope of paper					Only tissue with at least three intact villi included	Only tissue with at least three intact villi included	At day 4 in the no col group to obtain % lipid n=2 samples pooled	N=2 due to cost, one representative experiment shown	Only n=1 expt including FMT due to cost	Only n=1 expt including FMT due to cost	Only n=1 expt cohousing as continued with germ-free mice
